# Experiences of abortion: A narrative review of qualitative studies

**DOI:** 10.1186/1472-6963-8-150

**Published:** 2008-07-17

**Authors:** Mabel LS Lie, Stephen C Robson, Carl R May

**Affiliations:** 1 Institute of Health and Society, Newcastle University, William Leech Building, Newcastle upon Tyne, NE2 4HH, UK; 2 School of Surgical and Reproductive Sciences, Newcastle University, The Medical School, Framlington Place, Newcastle upon Tyne, NE2 4HH, UK; 3 Institute of Health and Society, Newcastle University, 21 Claremont Place, Newcastle upon Tyne, NE2 4AA, UK

## Abstract

**Background:**

Although abortion or termination of pregnancy (TOP) has become an increasingly normalized component of women's health care over the past forty years, insufficient attention has been paid to women's *experiences *of surgical or medical methods of TOP.

**Objective:**

To undertake a narrative review of qualitative studies of women's experiences of TOP and their perspectives on surgical or medical methods.

**Methods:**

Keyword searches of Medline, CINAHL, ISI, and IBSS databases. Manual searches of other relevant journals and reference lists of primary articles.

**Results:**

Qualitative studies (n = 18) on women's experiences of abortion were identified. Analysis of the results of studies reviewed revealed three main themes: experiential factors that promote or inhibit the choice to seek TOP; experiences of TOP; and experiential aspects of the environment in which TOP takes place.

**Conclusion:**

Women's choices about TOP are mainly pragmatic ones that are related to negotiating finite personal and family and emotional resources. Women who are well informed and supported in their choices experience good psychosocial outcomes from TOP. Home TOP using mifepristone appears attractive to women who are concerned about professionals' negative attitudes and lack of privacy in formal healthcare settings but also leads to concerns about management and safety.

## Background

Although abortion or termination of pregnancy (TOP) by clinical means is politically contentious in some countries (notably the US), in most developed countries it has become a normalized [1] component of women's health care [2] over the past forty years. For most of this period, TOP was a surgical procedure but since the mid-1990s, pharmaceutical developments (i.e. RU-486 also known as mifepristone, and methotrexate [3]), have made medical TOP possible. Clinical trials have established that medical TOP provides a clinical and cost effective alternative to vacuum aspiration for the early termination of pregnancy [4-8]. While a Cochrane systematic review highlighted inadequate evidence [9], a more recent systematic review concluded that the incidence of side effects in medical abortion was low [10]. Even so, mifepristone has only been approved in the US since September 2000, whereas the UK and Sweden have had more than a decade of experience of its use and it is approved for use in 14 European countries [11].

The emphasis on establishing clinical and cost effectiveness of medical versus surgical TOP means that less attention has been paid to women's *experiences *of the two methods. This paper goes some way towards filling that gap by providing a narrative review of qualitative studies of women's experiences of TOP and their perspectives on surgical or medical methods. Given the importance of this topic to policy and clinical practice around reproductive health, this is a surprisingly small body of literature, but it is highly heterogeneous and contextually specific.

## Methods

An initial scoping exercise established that the qualitative research literature was too heterogeneous to permit a systematic review of qualitative studies along the lines proposed by Dixon-Woods [12], or a theoretical qualitative meta-synthesis using the methods proposed by Sandelowski [13]. For this reason, a narrative review [14] was undertaken.

The review focused on the period 1998–2007 because it was during this period that medical TOP has become established in practice. The primary focus of the review is therefore on women's experiences of TOP, and this meant that other studies (for example qualitative studies of attitudes and moral considerations) were excluded. Studies included in the review were identified by keyword searches of Medline, Psychinfo, CINAHL, ISI, and IBSS databases. Keywords searched included 'abortion', 'terminat*, pregnan*', 'unplanned pregnancy', in combination with 'qualitative study', semi-structured, ethnograph* experiences', of which 'abortion experiences' yielded the most relevant material. Manual searches of other relevant journals (*Reproductive Health Matters*; *Health Care for Women International*; *Contraception*) and reference lists of primary articles found from initial searches were also conducted. These searches revealed four comparative qualitative studies of surgical versus medical TOP [15-18] of which three were conducted in the US and one in Latin America. A further 14 qualitative studies of women's experiences of TOP using either method were identified [19-32]. This is an extraordinarily small body of peer-reviewed research papers given the importance and contentiousness of the topic.

While many authors have observed that qualitative studies have important strengths in health policy and practice research [33,34] the studies included in this paper also have limitations that should be acknowledged. The most important of these are their small size and limited scope. Because this is not a systematic review and few articles were found, evaluations of methodological quality were not used to exclude papers from the study. However, it was accepted that non-probability sampling was employed and for ethical reasons, participants were self-selected. Even so, many studies provided insufficient socio-demographic information about their research participants and only nine acknowledged study limitations and recruitment biases [17,19,23-28,31]. In common with other narrative reviews of qualitative studies, this means that we do not seek to assess the ways that participant selection may have influenced results.

Discussion of ethnicity was virtually absent. Only one study [23], included participants who did not speak the dominant language in the country in which it was conducted, so that the views of migrant minority ethnic women were often not taken into account. While one study [28] recruited a significant proportion (two thirds) from minority ethnic communities, no attempt was made to explain their results on the basis of ethnicity.

Most studies recruited at clinical sites with the help of health professionals, others by advertising in public spaces (e.g. university, women's magazine) and snowballing [21,31,32] and the majority of studies interviewed single women from their late teens to their twenties. Only two studies interviewed participants prior to TOP [20,30]; two were longitudinal [24,35]; and two investigated the longer term effects of abortion [31,32]. Apart from two ethnographies [22,24] all studies collected data through semi-structured or in-depth interviews.

## Results

The review identified two groups of qualitative studies on TOP.

(i) Studies that focused on experiences of *medical *TOP, (n = 4, summarised in table 1) mainly in comparison with experiences of *surgical *TOP. Three of these studies were conducted in the US. These included a study embedded in the 1994–95 pre-legalisation clinical trials of mifepristone [15], and two studies of the home administration of mifepristone within the Abortion Rights Mobilisation Trials [16,17]. A further study on Latin American women's perspectives on medical TOP was not connected with assessing mifepristone [18].

**Table 1:** Summary of study characteristics: Medical compared with Surgical TOP

**Author & Date**	**Title of article**	**Country**	**Sample characteristics**	**Details**
Simonds et al 1998 [15]	Abortion, Revised: Participants in the U.S. Clinical Trials Evaluate Mifepristone	U.S.	n = 27 women n = 78 health care workers Age: Over 18	Recruitment from a clinical trial site Individual interviews Focus groups with health care workers at 17 sites. Interview duration: average of 15–20 mins, up to 45 mins Timing: about 2 weeks after TOP
Elul et al 2000 [16]	In-depth Interviews With Medical Abortion Clients: Thoughts on the Method and Home Administration of Misoprostol	U.S.	n = 22	Recruitment from a clinical trial site In-depth interviews Duration: 30 mins Timing: immediately after their follow-up visit of which 9 interviewed one week after TOP.
Fielding et al 2002 [17]	Having an Abortion Using Mifepristone and Home Misoprostol: A Qualitative Analysis of Women's Experiences	U.S.	n = 43 n = 30 Mean age: 26 Around 3/4 white and single, most worked full-time.	Recruitment from a clinical trial site Pre and post abortion open-ended questionnaires In-depth interviews Interview duration: 30 mins Timing: 1–6 weeks following medical abortion.
Lafaurie et al 2005 [18]	Women's Perspectives on Medical Abortion in Mexico, Colombia, Ecuador and Peru: A Qualitative Study	Latin America	n = 49 Age: most in their mid-20s. Women from urban and rural settings and range of socio-economic backgrounds	Recruitment through clinicians who provide both MTOP and STOP In-depth interviews in Spanish Duration:1–2 hrs

(ii) Studies that explored *general *experiences of TOP (n = 14, summarised in table 2). These focused on the process of arranging TOP [19,28], and the experience of undergoing it [21,22,26,30]. Two studies highlighted the influence of cultural and contextual features [23,24], with one looking more specifically at a sample of women involved in a clinical trial [25]. Other studies investigated the role of the male partner in TOP [35,36]; experiences of repeated TOP [20]; and recollections of abortion experiences years after undergoing the procedure [31,32]. Two studies specifically explored teenage TOP [24,29] and two the relationship between TOP and contraceptive service provision [29,37].

**Table 2:** Summary of study characteristics: general experiences of TOP

**Author & Date**	**Title of article**	**Country**	**Sample characteristics**	**Details**
Harden & Ogden 1999 [19]	Young women's experiences of arranging and having abortions	U.K.	n = 54 Age: 16–24. NHS and private patients.	Recruitment from two hospitals and two voluntary sector clinics Interview duration: 10–60 mins Timing: Interviews between 1–3 hours after their abortion
Tornbom & Moller 1999 [20]	Repeat abortion: a qualitative study	Sweden	n = 20 Age: 20–29 Women who had had between 1–5 abortions in the past 5 years.	Convenience sampling of women seeking abortion at a family planning unit. Duration: one and a half to two hours
McIntyre et al 2001 [21]	The Intersection of Relational and Cultural Narratives: Women's Abortion Experiences	Canada	n = 14 Age: 19–44 Wide range of social backgrounds. All Canadian-born, English-speaking and White.	In-depth interviews with women who had had an abortion were recruited from posters in one clinic and letters in handouts from another clinic
Bennett 2001 [22]	Single women's experiences of premarital pregnancy and induced abortion in Lombok, Indonesia	Indonesia	n= 116 Age: 16–25 Single mostly Muslim women from a variety of ethnicities and backgrounds	In-depth interviews (n = 35) Life story accounts (n = 15) Interviews with abortion providers (n = 8) 8 focus groups followed by workshops (n = 58 participants) Participation observation.
Remennick & Segal 2001 [23]	Socio-cultural context and women's experiences of abortion: Israeli women and Russian immigrants compared	Israel	n = 23 Israeli women n = 25 Russian immigrants Age: 20 or more. Immigrants: typically less fortunate and poorly integrated, Israeli women: a mix of middle-class and less advantaged.	Recruited at abortion clinics and through the Israeli FPA counselling services. Interviewed using semi-structured thematic guide Timing: within 3 months of surgical TOP.
Andrews & Boyle 2003 [24]	African American Adolescents' Experiences with Unplanned pregnancy and Elective Abortion	U.S.	n = 12 Age: 15–18 African American	Recruited through a non-profit family planning and abortion clinic. Focused ethnographic study. Interview duration: 1 to 1 1/2 hours Timing: On day of TOP followed by 2^nd ^and 3^rd ^interviews 6–8 months later 27 interviews in total. Data analysis triangulated with field notes, informal interviews and participation observation.
Fielding et al 2004 [25]	Social Context and the Experience of a Sample of U.S. Women Taking RU-486 (Mifepristone) for Early Abortion	U.S.	n = 35 Around 3/4 were white with some college education, and 85% were employed.	Recruited from a clinical trial site In-depth interviews, majority by telephone Duration: 30 minutes Timing: 1–6 weeks following their clinical follow-up visit
Alex and Hammarström 2004 [26]	Women's experiences in connection with induced abortion – a feminist perspective	Sweden	n = 5 Age: 19–33 Not highly educated	Recruited from one health centre In-depth interviews Duration: 50 – 140 mins | Timing: about one month after TOP
Kero et al 2004 [27]	Wellbeing and mental growth – long-term effects of legal abortion	Sweden	n = 61 Age: 28 (mean and median) 29 employed, 18 students	Follow-up study from a questionnaire survey in one hospital. Semi-structured telephone interviews Duration: 30–40 mins Timing: 4 months (61 women) and 12 months (58 women) post TOP
Kumar et al 2004 [28]	Decision making and referral prior to abortion: a qualitative study of women's experiences	U.K.	n = 21 Of varying ages and gestations, 14/21 from an ethnic minority community.	Recruited from three NHS TOP provider units (2 voluntary sector abortion providers). In-depth interviews Duration:1–2 hrs Timing: 3–9 weeks after TOP
Lee et al 2004 [29]	A matter of choice? Explaining national variations in teenage abortion and motherhood	U.K.	n = 103 Age: 17 and under at the time of pregnancy, from selected sites in England, Wales and Scotland.	Phase 3 of large national study: intensive interviews with 52 young mothers and 51 young women who experienced TOP, Timing: up to 9 years after event.
Hallden et al 2005 [30]	Meanings of Being Pregnant and Having Decided on Abortion: Young Swedish Women's Experiences	Sweden	n = 10 Age: 18–20	Recruited from three clinics in two cities. In-depth interviews Timing: 4–20 days before TOP
Trybulski 2005 [31]	Women and abortion: The past reaches into the present	U.S.	n = 16 Age: 38–92 European-American women with 12 or more years of education. All had had an abortion 15 years or more previously.	Convenience sampling through flyers in public places, an advertisement in a women's magazine, and through referrals. Interview duration: one and a half to two hours.
Goodwin & Ogden 2007 [32]	Women's reflections upon their past abortions: An exploration of how and why emotional reactions change over time	U.K.	n = 10 aged 23–31 Of mixed ethnicities Three had a medical abortion.	Recruitment through advertisements in a university. In-depth semi-structured interviews conducted in university premises. Duration: 20–90 minutes Experienced TOP 1–9 years previously.

Analysis of the results of studies included in this paper revealed three main themes: experiential factors that promote or inhibit the *choice *to seek TOP; *experiences *of TOP; and experiential aspects of the *environment *in which TOP takes place.

### 1. Choices

The watchword of campaigners for abortion services has been that it is the woman's right to freely choose between abortion and pregnancy [38]. Studies reviewed for this paper suggest that although moral values are important [15,21,26,27], the choice to seek TOP is a pragmatic one that reflects the impact of pregnancy and childbearing on personal and household circumstances [17,18,21,26,27,29]. A number of studies described the role male partners played in women's decision of whether to undergo the procedure [16,20-22,24,30]. Lone mothers are often economically disadvantaged, but in Sweden, where universal childcare provision makes lone parenthood economically viable, one study showed that participants (n = 5) preferred not to bring up children on their own [26]. Partnered or married women were also concerned about planning their families well [27], taking into consideration their partners' attitudes and the needs of their children [18] and their quality of life [17]. However, a U.S. study reported that women were more likely to confide in their female friends about their pregnancy than family members or partners [25]. Women's childhood experiences such as growing up in a broken home could also affect women's decisions [26]. Studies conducted with women under the age of 21 revealed that other factors such as immaturity, parental attitudes, and education and employment prospects were more important than moral considerations [24,29,30]

Whatever women's circumstances, studies in this review suggest that the decision to seek TOP usually precedes any encounter with heath care professionals [17,28,29]. However, such decisions are moderated by the value systems and social norms of the society or community in question [15,19,22-24,29]. Feelings of ambivalence in the decision-making process were highlighted in a Swedish study [26], where women felt positive towards the right to abortion, but negative about their own decision to abort. It is argued that TOP allows women to return to 'normality' psychologically, physiologically and socially, and women appreciated being treated in a non-stigmatised way [19]. However, a study conducted in the UK found that the majority of teenage mothers who were interviewed did not associate motherhood with lack or loss of opportunity [29].

The range of services available also affects the choices of women. However, papers identified for review provided little about how the *choice of TOP provider *is framed, or even what choices are available. One study of young women in the UK [29] found that they preferred family planning services rather than general practitioners for their first point of contact and referral, for reasons of greater anonymity and specialised treatment. Anonymity and confidentiality are key issues in all settings where TOP is stigmatised [21-24]. For this reason, Israeli women tended to avoid publicly subsidised formal procedures opting instead for private abortion providers [23].

In the UK, expectations of better personal treatment and confidentiality were also reasons why some women chose private or voluntary sector clinics over National Health Service (NHS) clinics, although cost is an issue [19]. Those who had used independent providers reported more positive experiences than those who had used the NHS [29]. Further evidence of this comes from another British study [28], where participants (n = 21) reported difficulties in getting an urgent appointment with their family doctor, problems with the NHS telephone booking system and being asked by doctors to further consider their decision, thus delaying the process.

Finally, the *choice of method *is dependent not only on service availability but on medico-legal considerations such as the gestational age. Once again, data on this topic are very limited. Pragmatic reasons such as effectiveness and the side effects were found to over-ride women's moral and political considerations in one US study [17]. Previous experiences of surgical abortion may have led women to seek medical rather than surgical TOP in two other US studies [16,17]. The experiences of other family members or friends who had undergone abortion can also be influential [17,30].

### 2. Experiences

Studies that concentrated on women's experiences of the TOP procedure prefaced their findings with an account of the specific medical regimens in place at the time of the study. The US studies focused on women's perceptions of medical abortion as a new procedure, and often compared this with surgical TOP. In this context, women identified medical abortion as a way to avoid surgery, and anaesthesia and that permitted them privacy, autonomy and a greater sense of control [15,17-19]. Simonds *et al *[15], in particular, explored the idea of abortion being 'natural' describing this as 'not-really-abortion, but rather as a late period that finally comes' (p1316). As such, medical TOP was associated with reduced feelings of guilt for some participants in her study. This 'naturalness' (a subjective association with a miscarriage or menstruation without the insertion of instruments) seems to outweigh the pain and prolonged nature of the procedure, including the sight of fetus. Other women focused on the pain as a necessary part of the process [16].

Complex emotional experiences appear to be integral to TOP. These include regret and guilt [17,22], distress and anxiety [17,22,27] and grief, loss, emptiness and suffering [21]. These experiences are related to gestational age, for example, in one study a medical termination before any symptoms of pregnancy were perceived was described as involving a 'loss' whereas a surgical termination was described as a 'death' [16]. Anxiety about sterility and death is also experienced by some women [16,18,26]. Women were also found to associate an abortion with taking responsibility [27] for the consequences of what they considered was an irresponsible act [19], especially in medical TOP where women were conscious during the procedure [15]. Another study [16] described the experience of a medical abortion as a chance to grieve, and the pain experienced was described by the authors as 'cathartic', one woman describing this as 'a personal investigation into your own pain' (p171).

Such perceptions are mediated by the moral context within which the women are located. In Indonesia, for example, women's perception of the fetus is influenced by the Islamic view that ensoulment takes place at 120 days of pregnancy [22]. In the US, Pro-Life argument against TOP is rich with images of a destructive, act, often explicitly called *murder*, leading some women to think that they 'killed a baby', but also realising 'it wasn't really a child' [17]. In a study by Fielding and Schaff [25], reservations about abortion in the second trimester onwards were unanimous except in relation to abnormalities. The language used to describe the fetus reflects the closeness or distance that women feel towards the life growing in their bodies and impacts on women's post-abortion emotional reactions [25,30,32]. In one trial, women were encouraged to look at the expelled fetus at home, but the authors say that 'dramatic' responses were rare [15]. Some women in this study described relief in not seeing a distinguishable human being when the fetus was expelled.

Feminist researchers provide insights into the interaction of TOP with notions of reproductive independence. A study [27] on the long term emotional effects of abortion found that more than half of the women who had reported both positive and painful feelings continued to report these feelings after 12 months. However, respondents reported they coped well, experiencing strengthened self-esteem, personal growth and maturity over the year. A study [30] of young Swedish women (n = 10) found that they encountered an understanding of themselves, their bodies, their fertility, and the meaning of adult motherhood. A study [24] of African-American adolescents (n = 12), aged between 15–18, highlighted their poor knowledge of reproductive processes and health and suggested that elective TOP was a 'positive, growth-enhancing experience' (p432), with participants being empowered by their experience of decision-making. However, Simonds *et al *[15] showed that in a clinical trial, medical abortion may have been perceived no less invasive as surgical abortion because of repeated insertions of pessaries, pelvic examinations, and ultrasound examinations, to ensure the success of the procedure.

Other studies highlight the isolation of women undergoing TOP and their concerns to conceal it from others [21,26]. In studies of the home use of misoprostol [17,18], there are accounts of women who undergo the abortion alone, or in secret with others such as family members around but unaware of the situation. In contrast, women in another clinical trial [16] described the active participation of partners or friends who helped to minimise their discomfort by rubbing their backs, bringing them tea, or monitoring their blood loss. Women with knowledge of how TOP works, and who have support from both their clinic and their partner seem more likely to experience a better outcome [18]. Women's cultural affiliations and beliefs also have a bearing on their emotional experiences [18,22,27]. For example, Israeli women tended to interpret abortion as a personal failure whereas Russian immigrants looked upon it as bad luck or a mistake [23]. In relation to the emotional impact of the abortion experience, a woman's preparedness and post-abortion support [32] as well as the emotional work required from nurses in family planning and abortion clinics [26] were important considerations.

### 3. Environment

The role of service providers is examined in most of the studies and British studies have focused especially on health services access and quality [19,28,29]. The process of seeking abortion in the UK is sometimes confusing because of inadequate information and extended because of delays in referrals. In three US studies [15-17] participants compared positive experiences of treatment by professionals providing medical TOP in clinical trials with professionals' negative attitudes and impersonal clinic settings in ordinary services. A Canadian study [21] identified a mismatch between women's normative expectations that health care providers should provide them with options and access to whatever medical services they might need, and what they perceived to be an unsympathetic reception from medical staff. The effect of such attitudes is assumed to discourage women from seeking abortion, but there is no systematic evidence to support this assumption. In an Israeli study, Russian immigrants objected to state interference into their choice to abort, but were impressed with the quality of publicly provided abortion services and sympathetic staff [23].

Women's experiences of patient care during an abortion are also affected by the method of termination. In US trials on medical TOP, women relied on health professionals to assure them about the safety of the new procedure and to determine if the termination had been successful [17]. Women needed more counselling from clinical staff about the procedure of medical termination [17]. This may reflect the need to assess if they were appropriate candidates for the procedure [15]. Women also had to be assured of ready access to medical information and help from clinical staff. In reports [15] of experiences with surgical TOP, treatment by medical staff figured more prominently than the actual physical experience of abortion.

In some contexts, the attitudes of health providers to abortion were relative to the marital status of the women [22]. In Indonesia for example, medical staff endorse abortions as a form of birth control for married women, but held disapproving attitudes towards pre-marital sex which impact on young women's feelings of guilt and shame. A study on teenage mothers in the UK [29] also reported doctors' disapproval. In the UK, clinical attitudes appear to be more negative towards the termination of pregnancies after the first trimester and some NHS clinics do not offer services for late abortions.

Studies that included primary care primary care professionals suggested that these were perceived as less sympathetic and supportive than professionals working in abortion services. The latter were perceived to be more caring and less judgemental [19,28]. This distinction was also found in one of the U.S. studies, although clinical trial staff were also perceived as more conscientious than women's usual health care providers.

Counselling is referred to in different ways in the studies but most particularly as counselling prior to the TOP to discuss the different methods, their benefits, what to expect, compliance and follow-up [17] and in relation to decision-making [28]. Other studies take a nursing perspective referring to the emotional work of nurses [26] and the importance of providing opportunities for women to express their suffering [27]. The importance of counselling is highlighted particularly where women had not told family or friends about their pregnancy [28]. However, unnecessary or superficial counselling has also been questioned [28,29]. In some parts of the non-Western world where women are more vulnerable, women's decision-making regarding abortion was influenced by the recommendations of the abortion provider and cost implications [18,22,23]. In most studies, information provision and knowledge were critical factors. An American study recommended that each patient be given a choice in the amount of information she receives, and information packs could be provided accordingly [17]. In relation to contraception however, knowledge needs to be integrated into practice for effective family planning [20]. The physical setting e.g. waiting rooms, and cold, unfamiliar wards was also referred to in some studies [19,26]. While some women appreciated the presence of other women in alleviating the loneliness of the experience, others were concerned about privacy and the risk of meeting someone they knew in the waiting room [21].

Some studies also investigated women's experiences of medical TOP at home rather than at a clinical facility [16-18,22]. In the US, Fielding [17] and Elul [16] identified familiar surroundings, privacy and not having to encounter strangers, as adding to women's appreciation of home TOP. However, there are situations in which home abortions are problematic, for example where the abortion needs to be kept hidden from the rest of the household because of shame [18,22]. This is particularly complicated where women are victims of domestic or sexual violence. Women also fear the risks of having an abortion at home where health professionals are not readily available to them.

## Conclusion

Qualitative studies published on TOP within the time frame of this review have been limited in scope and detail. In this article, we have identified two main groups of studies; those that specifically address the issue of medical abortion, and those that explore the experiences of TOP more generally. Studies reviewed in this paper were influenced by a range of contextual factors such as political, ethical, social and legislative environments as well as health, economic and welfare systems. Research from the US, UK and Sweden dominated the literature, but these three countries have very different patterns of service provision. This review leads to four main conclusions.

(i) Women's choices about whether, where, and how, TOP should be undertaken are mainly pragmatic ones that are related to negotiating finite household and psychosocial resources.

(ii) Rapid access to services characterised by supportive non-judgemental staff who delegate medical control over the process to women appear to characterise positive responses to medical TOP.

(iii) Home TOP using mifepristone appears attractive to women who are concerned about professionals' negative attitudes and lack of privacy in formal healthcare settings but also leads to concerns about management and safety.

(iv) Women who are well informed and supported in their choices experience good psychosocial outcomes from TOP.

These are broad conclusions derived from a very limited corpus of qualitative research. A recent review [39] of psychological studies of TOP identified discrepancies between societal and individual experiences, due to "theoretical and methodological deficiencies plaguing this area of study, with the available data often missing the complexity and depth of individuals' inner experiences" [37:238]. This is also true of many of the qualitative studies reviewed in this paper, suggesting that major opportunities to inform current policy and practice debates – utilizing the strengths of qualitative methods – have been missed.

## Conflict of interests

The authors declare that they have no competing interests.

## Authors' contributions

CRM conducted an initial literature scoping exercise. MLSL conducted the literature searches, collected and collated articles, and drafted this paper. SCR and CRM commented in detail on drafts and contributed to the final version of the manuscript.

## Pre-publication history

The pre-publication history for this paper can be accessed here:



## References

[B1] May C (2006). A rational model for assessing and evaluating complex interventions in health care. BMC Health Service Research.

[B2] Berer M (2005). Medical abortion: issues of choice and acceptability. Reproductive Health Matters.

[B3] Wiebe E, Dunn S, Guilbert E, Jacot F, Lugtig L (2002). Comparison of abortions induced by methotrexate or mifepristone followed by misoprostol. Obstet Gynecol.

[B4] Ashok PW, Hamoda H, Flett GMM, Kidd A, Fitzmaurice A, Templeton A (2005). Patient preference in a randomised study comparing medical and surgical abortion at 10-13 weeks gestation. Contraception.

[B5] Ashok PW, Kidd A, Flett GMM, Fitzmaurice A, Graham W, Templeton A (2002). A randomized comparison of medical abortion and surgical vacuum aspiration at 10-13 weeks gestation. Human Reproduction.

[B6] Creinin MD (2000). Randomized comparison of efficacy, acceptability and cost of medical versus surgical abortion. Contraception.

[B7] Harvey SM, Beckman LJ, Satre SJ (2001). Choice of and Satisfaction with Methods of Medical and Surgical Abortion among U.S. Clinic Patients. Family Planning Perspectives.

[B8] Henshaw R, Naji SA, Russell IT, Templeton A (1993). Comparison of medical abortion with vacuum aspiration: women's preferences and acceptability of treatment. British Medical Journal.

[B9] Say L, Kulier R, Gulmezoglu M, Campana A (2002). Medical versus surgical methods for first trimester termination of pregnancy.. Cochrane Database Syst Rev.

[B10] Zou Y, Li Y, Lei Z, Lu L, Jiang S, Li Q (2004). Side effect of mifepristone in combination with misoprostol for medical abortion. [Chinese]. Chung-Hua Fu Chan Ko Tsa Chih [Chinese Journal of Obstetrics & Gynecology].

[B11] Jones RK, Henshaw SK (2002). Mifepristone for Early Medical Abortion: Experiences in France, Great Britain and Sweden. Perspectives on Sexual and Reproductive Health.

[B12] Dixon-Woods M, Fitzpatrick R, Roberts K (2001). Including qualitative research in systematic reviews: opportunities and problems. Journal of Evaluation in Clinical Practice.

[B13] Sandelowski M, Docherty S, Emden C (1997). Qualitative metasynthesis: Issues and techniques. Research in Nursing & Health.

[B14] Collins JA, Fauser BCJM (2005). Balancing the strengths of systematic and narrative reviews. Hum Reprod Update.

[B15] Simonds W, Ellertson C, Springer K, Winikoff B (1998). Abortion, revised: participants in the U.S. clinical trials evaluate mifepristone. Social Science and Medicine.

[B16] Elul B, Pearlman E, Sorhaindo A, Simonds W, Westhoff C (2000). In-depth Interviews with Medical Abortion Clients: Thoughts on the Method and Home Administration of Misoprostol. J Am Med Womens Assoc.

[B17] Fielding SL, Edmunds E, Schaff EA (2002). Having an Abortion Using Mifepristone and Home Misoprostol: A Qualitative Analysis of Women's Experiences. Perspectives on Sexual and Reproductive Health.

[B18] Lafaurie MM, Grossman D, Troncoso E, Billings DL, Cháveze S (2005). Women's Perspectives on Medical Abortion in Mexico, Colombia, Ecuador and Peru: A Qualitative Study. Reproductive Health Matters.

[B19] Harden A, Ogden J (1999). Young women's experiences of arranging and having abortions. Sociology of Health and Illness.

[B20] Törnbom M, Möller A (1999). Repeat abortion: a qualitative study. Journal of Psychosomatic Obstetrics and Gynecology.

[B21] McIntyre M, Anderson B, McDonald C (2001). The Intersection of Relational and Cultural Narratives: Women's Abortion Experiences. Canadian Journal of Nursing Research.

[B22] Bennett LR (2001). Single women's experiences of premarital pregnancy and induced abortion in Lombok, Eastern Indonesia. Reproductive Health Matters.

[B23] Reminnick L, Segal R (2001). Socio-cultural context and women's experiences of abortion: Israeli women and Russian immigrants compared. Culture, Health and Sexuality.

[B24] Andrews J, Boyle J (2003). African American Adolescents' Experiences with Unplanned Pregnancy and Elective Abortion. Health Care for Women International.

[B25] Fielding SL, Schaff EA (2004). Social Context and the Experience of a Sample of U.S. Women Taking RU-486 (Mifepristone) for Early Abortion. Qual Health Res.

[B26] Aléx L, Hammarström A (2004). Women's experiences in connection with induced abortion - a feminist perspective. Scandinavian Journal of Caring Services.

[B27] Kero A, Högberg U, Lalos A (2004). Wellbeing and mental growth—long-term effects of legal abortion. Soc Sci Med.

[B28] Kumar U, Baraitser P, Morton S, Massil H (2004). Decision making and referral prior to abortion: a qualitative study of women's experiences. Journal of Family Planning and Reproductive Health Care.

[B29] Lee E, Clements S, Ingham R, Stone N (2004). A matter of choice? Explaining national variations in teenage abortion and motherhood.

[B30] Halldén BM, Christensson K, Olsson P (2005). Meanings of Being Pregnant and Having Decided on Abortion: Young Swedish Women's Experiences. Health Care for Women International.

[B31] Trybulski JA (2006). Women and abortion: the past reaches into the present. Journal of Advanced Nursing.

[B32] Goodwin P, Ogden J (2007). Women's reflections upon their past abortions: An exploration of how and why emotional reactions change over time. Psychology and Health.

[B33] Leys M (2003). Healthcare policy: Qualitative evidence and health technology assessment. Health Policy.

[B34] Mays N, Pope C (1996). Qualitative research in health care.

[B35] Kero A, Lalos A (2004). Reactions and reflections in men, 4 and 12 months post-abortion. Journal of Psychosomatic Obstetrics & Gynecology.

[B36] Kero A, Lalos A, Högberg U, Jacobsson L (1999). The male partner involved in legal abortion. Human Reproduction.

[B37] Kumar U, Baraitser P, Morton S, Massil H (2004). Peri-abortion contraception: a qualitative study of users' experiences. Journal of Family Planning and Reproductive Health Care.

[B38] Oakley A (1986). Telling the truth about Jerusalem.

[B39] Coleman P, Reardon DC, Strathan T, Cougle JR (2005). The psychology of abortion: A review and suggestions for future research. Psychology and Health.

